# Taming the Appetite

**Published:** 1875-12

**Authors:** 


### TAMING THE APPETITE.

It has long been known by thoughtful physiologists that the use of meats and condiments as food encourage the appetite for strong drink. Instances innumerable go to prove that the desire for wine and stronger alcohols ceases, or becomes entirely managable on the adoption of a diet of fruits and cereals and vegetables. We are strongly of the opinion that the disuse of stimulating food—among which all flesh is to be reckoned—will eradicate the alcoholic diathesis, and with it all strong desire for alcoholic beverages.

This case is in point. A man of good family, was brought up by his parents to imbibe whiskey as a daily drink. At manhood the practice was second nature. His food was largely meat. He cared nothing for fruit or vegetables or bread.

Each day he consumed from a pint to a quart of whisky. Of course he was never quite sober. At last he was induced to give up meat by a friend who believed this course would lead to the abandonment of the poison. He breakfasted on oatmeal-mush and dined on beans and peas. Good bread from whole-wheat flour was abundantly provided, and this was all the food which he saw on his own table. He asked for meat at first, but the cattle-disease was cited as the excuse for not having meat served. The whisky rations soon ceased, without one word from anyone, and now neither alcoholic drink nor meat is ever touched by this man. In fact, both are disliked. The appetite is restored to purity and a natural condition; his appearance, health, and strength are greatly improved.

Then there is the case of a famous chemist, who, from being greatly addicted to intemperate eating and drinking, became a strict vegetarian, and, in six weeks, lost all desire for meats and alcohol. His bad headaches left him at the same time, and his precarious health became perfect.

Heavy drinkers are apt to be heavy eaters. Several such have been brought to decency, in both directions, by first giving up meat.

There was a prominent politician of 60, who had been addicted for thirty-five years to intemperate habits, and his constitution was shattered. After an attack of delirium tremens, he was induced to adopt a farinaceous diet, which cured him in seven months. He was very thin, but his weight increased 28 lbs. Two sisters, members of a family noted for intemperate habits, adopted vegetarianism, and were cured in about a year. A clerk who had lost several situations through intemperance was cured by vegetarianism, and was taken back by an employer at a higher salary than he had ever received. A governess, aged 40, lost her situation through intemperance, and was cured by adopting farinaceous diet in nine weeks. Two military pensioners were cured in six months. Three old sailors were cured in like manner in the same period.

There are certain articles of diet which seem to be specially antagonistic to alcohol. Beans and peas, thoroughly cooked, stand at the head. The dried beans or peas, when used, should be soaked in warm water twenty-four hours, and then boiled from one to three days. Onions and celery may be boiled with them to excellent advantage. If these vegetables are used green, they should also be cooked several hours, and not simply scalded as is not unusual. In fact, the usual cooking methods for all vegetables must be greatly modified. Nothing is done enough; nothing is properly done, as a rule. The oatmeal wheaten grits and mushes, all good, must receive from four to ten times as much cooking as they ordinarily get. All such foods must be cooked in a double boiler, so that no burning will be possible. No salt should be put in the article cooked, but the water lining, which protects the boiler, should be well salted, so as to carry the heat to a high point.

Whole-wheat flour bread is the best diet, and it should be so made and baked that a good deal of mastication is needed to prepare it to be swallowed. The harder it is, the better, if time is taken in its mastication. We have never yet happened to see a person who would assert for himself or herself that the disuse of stimulating food and alcohol had worked an injury to health. We think meat should be excluded from the diet of all patients at inebriate asylums, and that no stronger animal food than milk and eggs, with good grain foods, and fruits, and vegetables, are needed in health or in sickness, ordinarily.

---

WHOLE WHEAT FLOUR.

